# Computational Trials: Unraveling Motility Phenotypes, Progression Patterns, and Treatment Options for Glioblastoma Multiforme

**DOI:** 10.1371/journal.pone.0146617

**Published:** 2016-01-12

**Authors:** Fabio Raman, Elizabeth Scribner, Olivier Saut, Cornelia Wenger, Thierry Colin, Hassan M. Fathallah-Shaykh

**Affiliations:** 1 The University of Alabama, Birmingham, Department of Biomedical Engineering, Birmingham, Alabama, United States of America; 2 The University of Alabama, Birmingham, Department of Mathematics, Birmingham, Alabama, United States of America; 3 The University of Bordeaux, Department of Mathematics, Talence, France; 4 Universidade de Lisboa, Faculdade de Ciências da Universidade de Lisboa, Institute of Biophysics and Biomedical Engineering, Lisboa, Portugal; 5 The University of Alabama, Birmingham, Department of Neurology, Birmingham, Alabama, United States of America; University of Michigan School of Medicine, UNITED STATES

## Abstract

Glioblastoma multiforme is a malignant brain tumor with poor prognosis and high morbidity due to its invasiveness. Hypoxia-driven motility and concentration-driven motility are two mechanisms of glioblastoma multiforme invasion in the brain. The use of anti-angiogenic drugs has uncovered new progression patterns of glioblastoma multiforme associated with significant differences in overall survival. Here, we apply a mathematical model of glioblastoma multiforme growth and invasion in humans and design computational trials using agents that target angiogenesis, tumor replication rates, or motility. The findings link highly-dispersive, moderately-dispersive, and hypoxia-driven tumors to the patterns observed in glioblastoma multiforme treated by anti-angiogenesis, consisting of progression by Expanding FLAIR, Expanding FLAIR + Necrosis, and Expanding Necrosis, respectively. Furthermore, replication rate-reducing strategies (*e.g*. Tumor Treating Fields) appear to be effective in highly-dispersive and moderately-dispersive tumors but not in hypoxia-driven tumors. The latter may respond to motility-reducing agents. In a population computational trial, with all three phenotypes, a correlation was observed between the efficacy of the rate-reducing agent and the prolongation of overall survival times. This research highlights the potential applications of computational trials and supports new hypotheses on glioblastoma multiforme phenotypes and treatment options.

## Introduction

Glioblastoma multiforme is associated with a poor prognosis. Clinical trials of bevacizumab, a humanized anti-vascular endothelial growth factor monoclonal antibody, showed no significant effects on the overall survival times of patients with newly diagnosed glioblastoma multiforme [1–3]. A prospective randomized phase 3 trial of patients with newly diagnosed glioblastoma multiforme, after completion of concomitant chemoradiotherapy, to receive either adjuvant temozolomide chemotherapy alone, or temozolomide with Tumor Treating Fields, was recently completed. The data revealed that patients treated with Tumor Treating Fields had a longer overall median survival of 20.5 months vs 15.6 months in the temozolomide alone group [4]. Nowosielski et al. reported four types of glioblastoma multiforme progression patterns in patients treated by bevacizumab [5]. They distinguished each type according to radiologic progression differences in magnetic resonance imaging T1-weighted sequences and T2-hyperintense signals. Using similar criteria, we have reported an additional progression pattern in bevacizumab-treated patients; the latter is supported by the mathematical model used in this investigation [6]. Clinical data revealed that the progression patterns are associated with significant differences in overall survival times of glioblastoma multiforme patients treated by bevacizumab [5, 6]; specifically, patients that recur by expanding fluid-attenuated inversion recovery signal (FLAIR) have a significantly longer overall survival times.

The invasion by glioblastoma multiforme cells into the brain causes significant neurological morbidity. The motility of glioblastoma multiforme cells is believed to be mediated by two mechanisms. The first, concentration-driven motility, drives invasive cells from high to low concentrations; its rate being variable between tumors [7, 8]. The second mechanism, hypoxia-driven motility, is associated with increased motility under hypoxic conditions. Keunen *et al*. studied glioblastoma multiforme xenografts in animal brains and showed that treatment with bevacizumab lowered blood supply but was associated with an increase in infiltrating tumor cells [9]. Tang *et al*. reported that 4 out of 8 glioblastoma cell lines exhibit a phenotype of low-oxygen-induced accelerated brain invasion mediated by activation of c-src and neural Wiskott-Aldrich syndrome protein [10]. The oxygen threshold that controls the phenotypic switch is higher than what is typically anticipated for cancer-related hypoxia (*ie* 0.3%-1%); the enhancement in motility is observed at 5% as well as 1% ambient oxygen. Plasswilm *et al*. showed that hypoxia significantly increases motility of a glioblastoma cell line in an *in vivo* chicken model [11]. In addition, the results of Baker *et al*. demonstrate that, in the absence of angiogenesis, glioblastoma multiforme cells migrate towards existing normal microvessels and grow in the perivascular spaces [12]. All of these findings support the need to incorporate hypoxia-driven motility into models of glioblastoma multiforme growth with the goal of better understanding its effects on tumor growth and response to bevacizumab.

We have recently reported a mathematical model of glioblastoma multiforme growth and invasion at the scale of clinical magnetic resonance imaging that includes both concentration-driven and hypoxia-driven motility [6]. Here, we attempt to replicate the progression patterns of bevacizumab-treated glioblastoma multiforme and to test the hypothesis that the motility phenotype (*i.e*. concentration-driven vs hypoxia-driven) determines the progression pattern and predicts the overall survival times of bevacizumab-treated patients. We conduct a computational trial; the results support the hypothesis and define three motility-based phenotypes, highly-dispersive, moderately-dispersive, and hypoxia-driven tumors. Computational trials are also utilized to evaluate the therapeutic efficacy of rate- (*e.g*. Tumor Treating Fields) and motility-reducing agents in each phenotype and in a population study including all three. The findings suggest therapeutic avenues for each phenotype and correlate efficacy with overall survival times.

### Progression Patterns

Three magnetic resonance imaging indicators were used to distinguish among the four tumor progression patterns identified in [5] and [6]: (1) the gadolinium (Gad) enhancement visible on T1-weighted magnetic resonance imaging signals, (2) FLAIR/T2-weighted magnetic resonance imaging signals, and (3) necrosis. Nowosielski *et al*. have examined the magnetic resonance imaging scans of 83 patients prior to, during, and following resistance to treatment with bevacizumab and described four distinct progression patterns, which they labeled as: (1) primary nonresponder, (2) cT1 flare-up, (3) T2-diffuse, and (4) T2-circumscribed. T2 refers to the signal observed on FLAIR/T2-weighted magnetic resonance imaging, and cT1 refers to gadolinium enhancement observed on T1-weighted magnetic resonance imaging. The model in [6] uncovered an additional pattern of glioblastoma multiforme progression by expanding necrosis and expanding FLAIR. The T2-diffuse and T2-circumscribed progression patterns from [5] are termed Expanding FLAIR and Expanding Necrosis, respectively. The progression pattern identified in [6] is Expanding FLAIR + Necrosis. Table 1 provides a summary of the nomenclature used to describe these radiologic progression patterns in both this paper and in the literature.

**Table 1:** Tumor motility phenotypes.

Tumor Motility Phenotype	Progression Pattern		Motility Type	
	Our Nomenclature	Literature Nomenclature	CoD	HypD
Highly-Dispersive	Expanding FLAIR	T2-Diffuse	High	High/Low
Moderately-Dispersive	Expanding FLAIR + Necrosis		Moderate	High/Low
Hypoxia-Driven	Expanding Necrosis	T2-Circumscribed	Low	High

Summary of the tumor motility phenotypes, the associated nomenclature in the literature and in this paper for the progression patterns of glioblastoma multiforme under anti-angiogenesis treatment, and the types of motility (hypoxia-driven, HypD, versus concentration-driven, CoD) that produce these phenotypes.

Present knowledge of glioblastoma multiforme biology and its response to bevacizumab offers an explanation for the mechanisms of the progression pattern of primary nonresponse and cT1 flare-up. The tumor is inherently resistant to targeting vascular endothelial growth factor in primary nonresponders, leading to an increase or no change in the gadolinium enhancement and FLAIR signals. In addition, the tumor acquires resistance over time when progression occurs by cT1 flare-up, or an increase in Gad, indicating new tumor mass. These two modes of tumor progression have also been described in the literature as “intrinsic non responsiveness” and “adaptive evasion”, respectively [13, 14]. Thus, the three glioblastoma multiforme tumors whose mechanisms of progression are being studied are: (1) Expanding FLAIR + Necrosis (2) Expanding FLAIR (T2-diffuse), and (3) Expanding Necrosis (T2-circumscribed). Table 2 summarizes the radiologic characteristics of the progression of each tumor under anti-angiogenesis treatment.

**Table 2:** Recurrence patterns.

Pogression Pattern	1st FU Gad	1st FU FLAIR	1st FU Necrosis	Progression Gad	Progression FLAIR	Progression Necrosis
**Expanding FLAIR + Necrosis**	↓	↓ or ↔	↑ or ↔	↔	↑	↑
**Expanding FLAIR**	↓	↑ or ↔	↔	↔	↑	↔
**Expanding Necrosis**	↓	↓ or ↔	↑ or ↔	↔	↔	↑

1st Follow-up (FU) describes magnetic resonance imaging appearance following initiation of anti-angiogenesis treatment as compared to pre-treatment magnetic resonance imaging appearance. Progression describes magnetic resonance imaging appearance at tumor progression as compared to the 1st Follow-up magnetic resonance imaging appearance. ↑ indicates an increase, ↓ a decrease, and ↔ no change. Gad refers to the volume of gadolinium-enhancement on magnetic resonance imaging of the brain.

#### Expanding FLAIR + Necrosis

Simulations of the model in [6] under anti-angiogenesis treatment revealed that glioblastoma multiforme continues to grow in silico by expanding necrosis and FLAIR (see Table 2). To validate the pattern of glioblastoma multiforme progression suggested by these simulations, we reviewed the magnetic resonance imaging of 69 patients diagnosed with glioblastoma multiforme and one patient with gliosarcoma, were treated with bevacizumab at first recurrence (see [6]). 23/70 patients met the criterion of at least one stable magnetic resonance imaging following the maximal effects of bevacizumab. These criteria were exceeding 25% reduction in gadolinium enhancement, indicating initial response to treatment. Subsequent images of 11/23 patients demonstrated expanding areas of both necrosis and FLAIR in the absence of or without new significant gadolinium enhancement. Since the area of FLAIR extended beyond the area of necrosis into healthy brain tissue, we refer to this progression pattern as Expanding FLAIR + Necrosis.

#### Expanding FLAIR

Of the 83 patient magnetic resonance imaging evaluated, Nowosielski *et al*. identified 15 with the T2-diffuse progression pattern. In order to correctly evaluate each progression pattern, they obtained baseline scans at median 14 days before treatment and followup scans at median 12 weeks after treatment [5]. The T2-diffuse progression pattern is characterized by a complete decrease in T1-weighted contrast enhancement followed by a signal increase in the FLAIR/T2-weighted signals at progression referred to in this paper as Expanding FLAIR (Table 2). There is little or no expanding necrosis of the brain associated with this progression pattern, and any new necrosis following treatment is localized to the original site of the tumor mass.

#### Expanding Necrosis

In Nowosielski *et al*., 17 patients exhibited the T2-circumscribed progression pattern. In this case, the tumor responds initially with a decrease in the T1-weighted gadolinium enhancement but progresses with an increase in the FLAIR/T2-weighted signals (Table 2). Unlike Expanding FLAIR + Necrosis, however, the area of the T2 signal corresponds to that of the T1-weighted image, or closely circumscribes the site of expanding necrosis and/or tumor mass. This progression pattern is termed Expanding Necrosis since the FLAIR signal is localized in close proximity to the site of necrosis and does not extend deep into healthy brain tissue like that observed in Expanding FLAIR + Necrosis (see above).

The motivation in studying these three glioblastoma multiforme progression patterns is the significant differences in survival times associated with the progression patterns. For example, as found in [6], the Expanding FLAIR + Necrosis progression pattern predicts a poor prognosis. There was a significant difference in the average progression-free survival time of 178 days (or six months) for the 11 patients that recurred by Expanding FLAIR + Necrosis versus 333 days (or 11 months) for the group that did not exhibit this progression pattern (Log-Rank *p* = 0.0002) [6].

Expanding FLAIR is the best possible outcome for the patient treated with bevacizumab: Nowosielski *et al*. found that in patients exhibiting this progression pattern, the overall survival following the start of bevacizumab was 3 times that of primary nonresponders and patients that recur by Expanding Necrosis [5]. Specifically, patients with progression by Expanding FLAIR had a median overall survival time following treatment of 17.7 months as compared to 5.0 months for patients with Expanding Necrosis, and 4.9 months for primary nonresponder (Log-Rank *p* = 0.001) [5]. Hence, the progression pattern of Expanding Necrosis represents the worst prognosis for a glioblastoma multiforme patient. Of interest is the observation that the poor overall survival of patients that recur by Expanding Necrosis following treatment is equivalent to the overall survival time of primary nonresponders [5].

### Description of Motility Phenotypes

**Fig 1:**
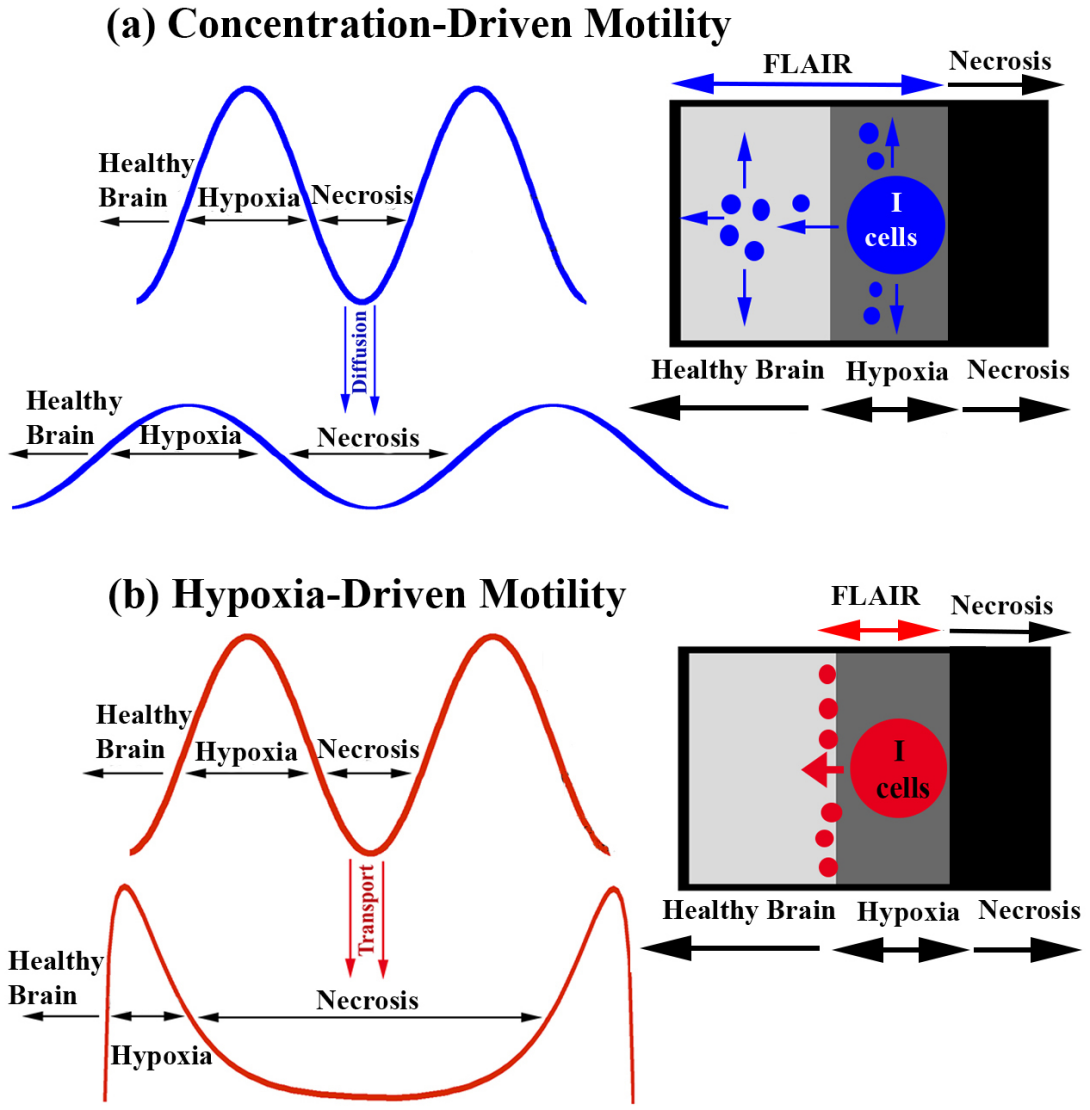
Dynamics of concentration-driven and hypoxia-driven motility. Cartoon depicting the differences between brain invasion by concentration-driven motility (a) and hypoxia-driven motility (b). The latter is triggered by necrosis and causes invasion and accumulation of *I* cells within a short distance of the tumor/healthy brain boundary. Concentration-driven motility causes dispersion of tumor cells away from the high density tumor mass such that the depth of invasion into healthy brain tissue is positively correlated with the intrinsic migratory ability of the tumor.

This model includes two tumor cell types: invasive cells and proliferative cells. The latter replicate, but do not move. However, in the presence of hypoxia, proliferative cells revert to invasive cells, which can move using hypoxia-driven and/or concentration-driven motility. These features are consistent with the go-or-grow phenotype. Fig 1 depicts the primary differences between invasive cell movement driven by hypoxia verses concentration. The left side of the figure depicts the time evolution of a one-dimensional slice of invasive cells driven by concentration alone (Fig 1a) and hypoxia alone (Fig 1b), while the right side shows a two-dimensional cartoon of differences in invasive cell motility along the hypoxic edge of the tumor for concentration-driven verses hypoxia-driven motility.

In contrast to concentration-driven motility, which acts on tumor motility by driving invasive cells in all directions away from higher concentrations of invasive cells towards lower concentrations, hypoxia-driven motility is sensitive to hypoxia, driving invasive cells unidirectionally away from brain necrosis and towards healthy oxygenated brain tissue (Table 3). In both cases, invasive cells are unable to move in the direction of the necrotic core. However, note the multi-directional movement (blue arrows) in the illustration for concentration-driven motility as opposed to the unidirectional movement (red arrow) in that of hypoxia-driven motility in Fig 1. Also, invasive cells are dispersed throughout healthy brain tissue in the concentration-driven motility model (see double-sided blue arrow labeled FLAIR) whereas they accumulate sharply at the edge of hypoxia and healthy brain tissue in the hypoxia-driven motility model (see double-sided red arrow labeled FLAIR).

**Table 3:** Hypoxia-driven versus concentration-driven motility.

	Movement in Tumor Mass	Movement in Healthy Brain	Driving Force	Necrotic Area	Other Names
**Concentration-Driven Motility**	High to Low Invasive Cell Concentrations	High to Low Invasive Cell Concentrations	Invasive Cell Concentration	Decreased Motility	•Passive Diffusion •Diffusion
**Hypoxia-Driven Motility**	Away from Necrosis Towards Healthy Brain	Does Not Move	Necrosis	Away From	•Active Transport •Directed/Active Migration •Dispersion •Haptotaxis

Summary of the key features distinguishing invasive cell movement by hypoxia-driven verses concentration-driven motility.

These different motility types largely affect the behavior of the tumor in our simulations and may offer explanations for the different progression patterns described in the sections above. In fact, we will show that tumor motility can be linked to the progression patterns (see Table 1). Table 3 summarizes the main differences between hypoxia-driven and concentration-driven motility. In another mathematical model of glioblastoma multiforme, the authors incorporate a motility term similar to our hypoxia-driven motility term for invasive cell movement, which they call haptotaxis and describe as “active” or “directed” migration (see [15]). For this reason, Table 3 also includes common names used in the literature to describe the two types of invasive cell movement (hypoxia-driven and concentration-driven).

### Clinical and Biological Questions

Given the distinctions between hypoxia-driven and concentration-driven motility and the unexplained tumor behavior with the three progression patterns described above, we set out to use our model to address the following clinical questions:

1. What are the mechanisms driving the three progression patterns: Expanding FLAIR + Necrosis, Expanding FLAIR, and Expanding Necrosis?
2. Why do patients with Expanding Necrosis have such low overall survival times equivalent to those observed in non-responders?
3. Are rate- and motility-reducing agents effective in any subgroups of glioblastoma multiforme?
4. What is the relationship between the efficacy of a therapeutic agent and the overall survival times?

Present clinical and biological methods and data are unable to answer these questions. However, our mathematical model is a unique tool fit to address these questions. We hypothesized that the rates and mechanisms of motility determine the progression patterns and predict the overall survival times. In the sections that follow, we present the materials and methods used to obtain the results of this investigation. We demonstrate that with a fixed replication rate for proliferative cells, we can simulate the three progression patterns (Expanding FLAIR, Expanding Necrosis, Expanding FLAIR + Necrosis) by changing *only* the rates of hypoxia-driven and concentration-driven motility of invasive cells. Using the hypoxia-driven and concentration-driven motility parameter choices needed to generate each progression pattern, we conduct computational trials that include each progression pattern as well as a control group of untreated tumors; the results of our trial reproduce the differences in overall survival times reported in [6] and [5]. We also apply computational trials to: 1) investigate the therapeutic benefits of rate- and motility-reducing agents in the three glioblastoma multiforme phenotypes, and 2) design a computational population trial that includes all three phenotypes as well as tumors with variable replication rates.

Not only does the mathematical model replicate the three progression patterns, but the titrations of hypoxia-driven and concentration-driven motility also reveal novel insights into the behavior of glioblastoma multiforme treated by anti-angiogenesis therapy. We use the underlying characteristics of hypoxia-driven and concentration-driven motility in our model to propose hypotheses regarding the mechanisms of tumor progression during anti-angiogenesis therapy. The simulation results also suggest therapeutic modalities for each of the phenotypes of glioblastoma multiforme and reveal a relationship between efficacy and overall survival. In particular, tumors with moderate and high concentration-driven motility may respond to rate-reducing agents like Tumor Treating Fields, while patients with hypoxia-driven glioblastoma multiforme may benefit from motility-reducing agents.

## Results

### Motility Phenotypes Determine The Progression Patterns in Anti-angiogenic Therapy

**Fig 2:**
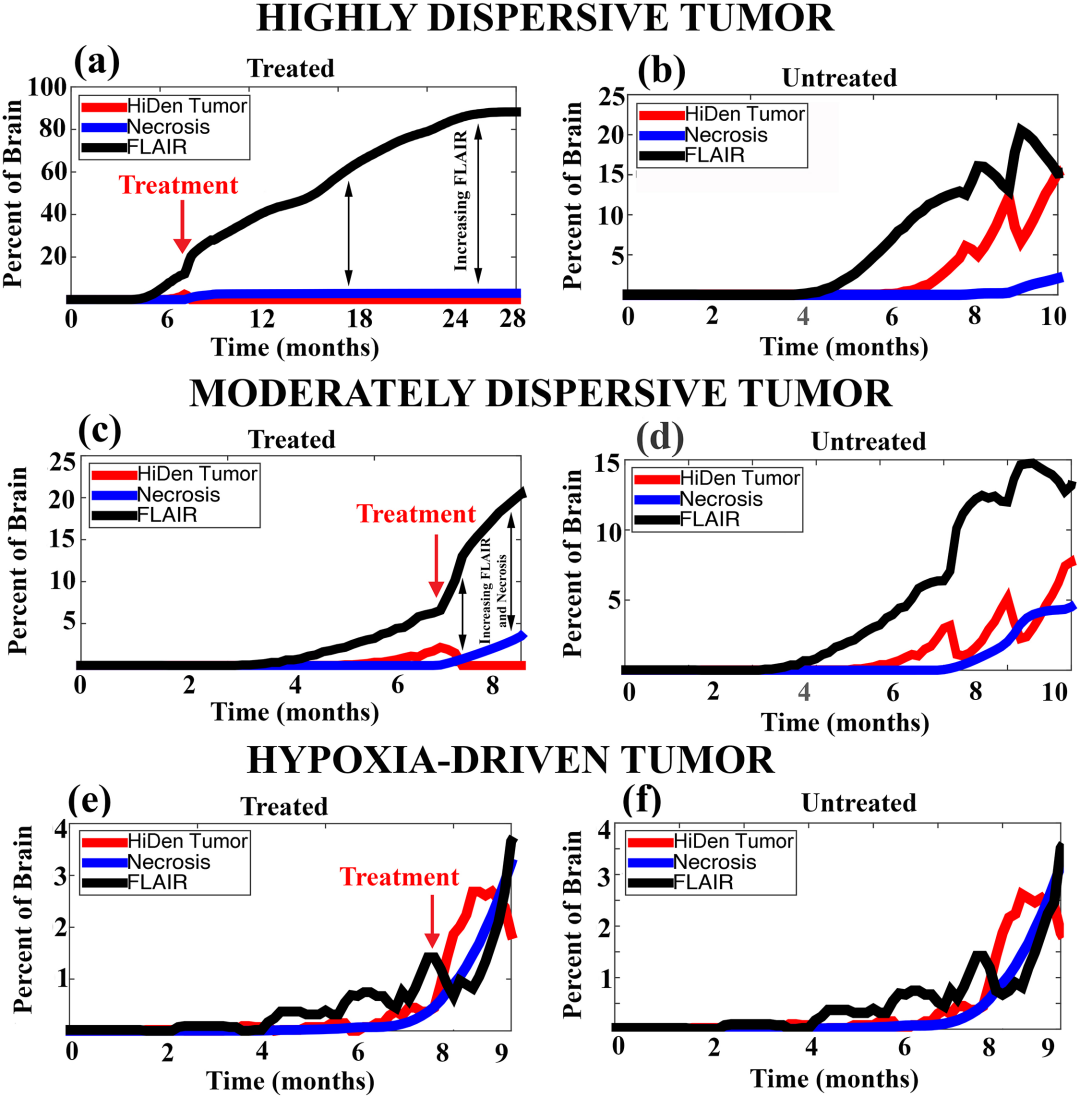
Progression curves by motility phenotypes for glioblastoma multiforme treated and not treated by bevacizumab. Each plot displays the time evolution of the percent of the brain occupied by invasive cells (FLAIR, black curve), high density (HiDen) tumor mass (red curve), and necrosis (*i.e*. 80% or more brain death, blue curve) for treated (a, c, and e) and untreated (b,d, and f) tumors. Red arrows denote the time of treatment. Double-sided black arrows emphasize the expanding difference in percent FLAIR and percent necrosis in the treated highly-dispersive and moderately-dispersive models. Notice the closeness of the FLAIR and necrosis curves in hypoxia-driven tumors treated by bevacizumab (e). (a), (c) and (e) correspond to the progression pattern of Expanding FLAIR, Expanding FLAIR + Necrosis, and Expanding Necrosis, respectively. The parameter choices for highly-dispersive, moderately-dispersive, and hypoxia-driven tumors in these simulations correspond to the high concentration-driven/high hypoxia-driven, moderate concentration-driven/high hypoxia-driven, low concentration-driven/high hypoxia-driven, respectively (see Table 4).

**Fig 3:**
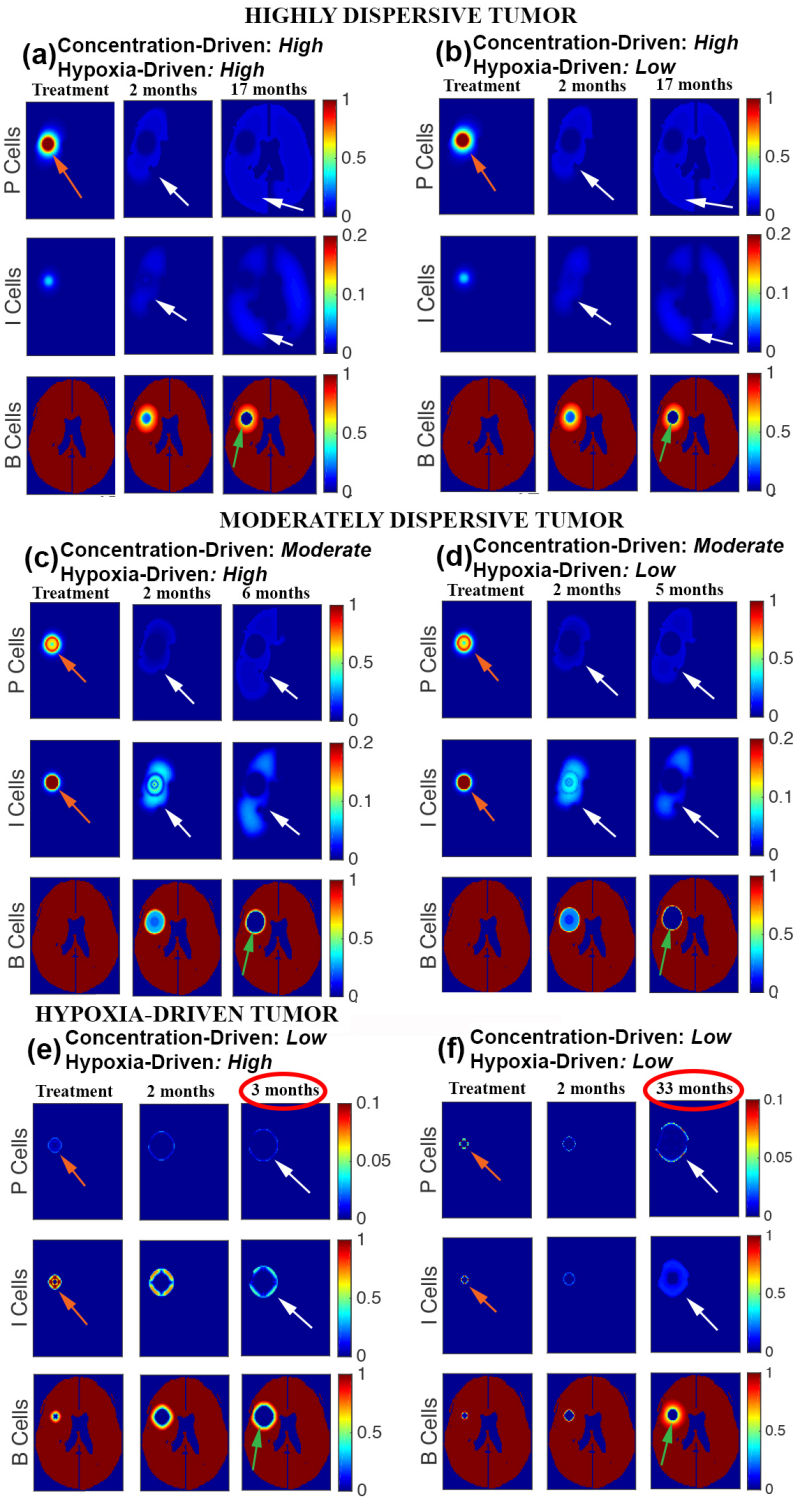
Progression patterns by motility phenotypes. Virtual magnetic resonance imaging of simulations reproducing progression by Expanding FLAIR (a and b), Expanding FLAIR + Necrosis (c and d), and Expanding Necrosis (e). Proliferative cells (P) show the size of the proliferating tumor, invasive cells (I) show the location of invasive cells, and B cells show normal brain cells and the location of necrosis (green arrows). In each simulation, the first time shot (treatment) is taken immediately prior to anti-angiogenesis treatment, the second time shot shows the 2-month follow-up, and the final time shot displays tumor appearance at the simulated time of death. Red arrows point to high-density proliferative cells. White arrows point to invasive cells and low-density proliferative cells in treated models. The tumor size at the start of treatment is as follows: 2.4% of the brain for (a) and (b), 2.6% of the brain for (c) and (d). For simulations (e) and (f), the tumor sizes are 1.1% for (e) and 0.08% for (f) with areas having 80% or more necrosis at the start of treatment are 0.3% and 0.4% of, respectively. The percents of the brain with 80% or more necrosis at time of death are: 1.5% (a and b), 4.2% (c and e), 5.0% (d), 1.3% (f). Finally, the percentages of brain occupied with FLAIR at the end of the simulations are: 84% (a and b), 26% (c), 23% (d), 6% (e), and 11% (f). The corresponding parameter choices for hypoxia-driven and concentration-driven motility may be found in Table 4.

**Fig 4:**
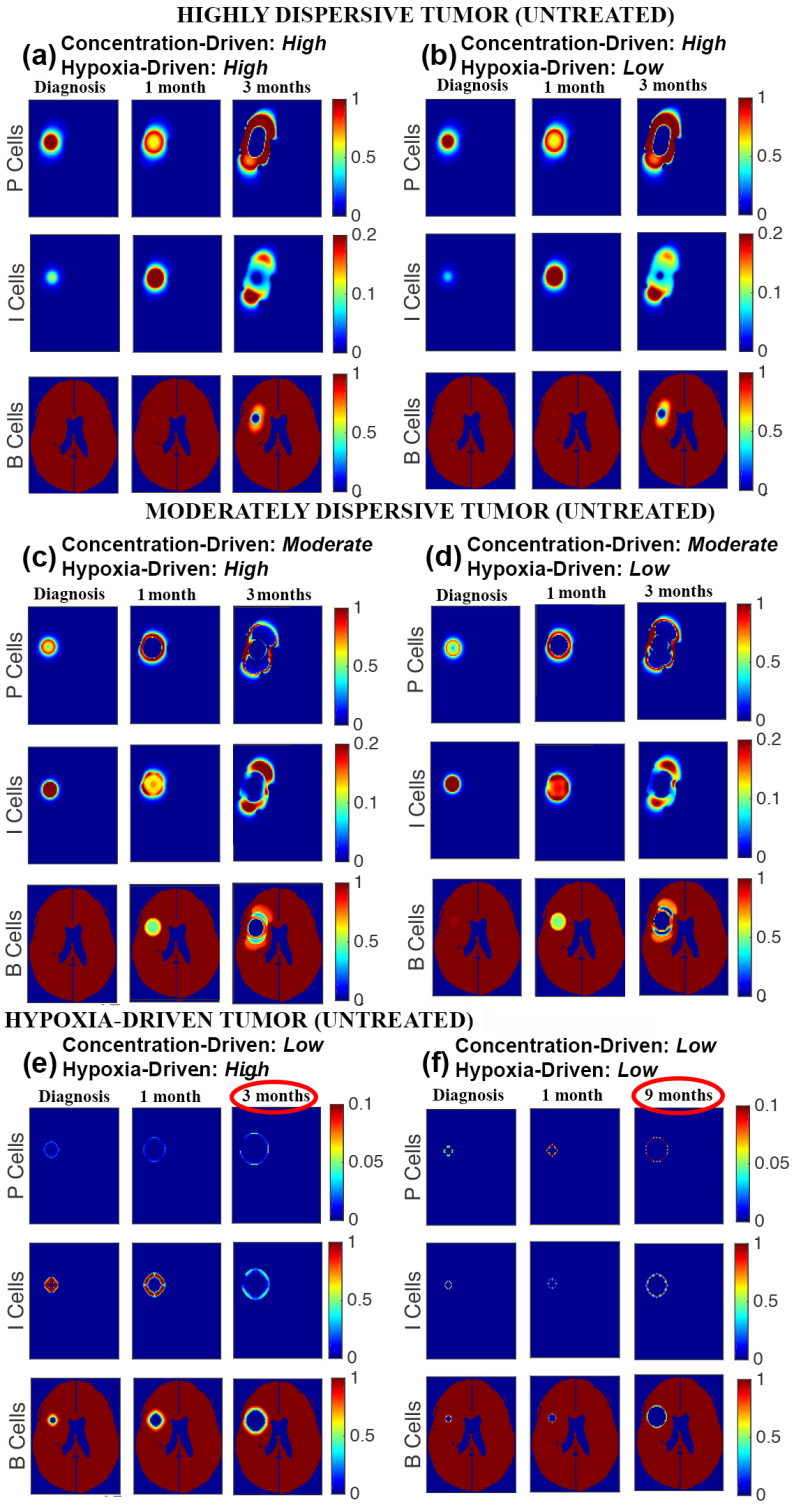
Tumor growth by motility phenotypes. Virtual magnetic resonance imaging of simulations of untreated tumor growth in highly-dispersive tumors (a and b), moderately-dispersive tumors (c and d), and hypoxia-driven tumors (e and f). In each simulation, the first time shot (diagnosis) is identical to the first time shot for the treated tumors in Fig 3, the second time shot shows first follow-up (1 or 2 months), and the final time shot displays tumor appearance at death. The percents of the brain with 80% or more necrosis at time of death are: 0.7% (a and b), 3.0% (c), 4.1% (e), 4.3% (d and f). Percents of brain with the presence of high density tumor at time of death are: 15% (a and b), 5% (c), 6% (d), and less than 0.3% (e and f). The corresponding parameter choices for hypoxia-driven and concentration-driven may be found in Table 4. P, I, and B refer to the proliferative, invasive, and brain cells, respectively.

The findings reveal that variations in the motility phenotypes/mechanisms (hypoxia-driven and concentration-driven) determine tumor response to anti-angiogenesis therapy. Table 4 displays the progression patterns observed, in silico, of several parameter choices for hypoxia-driven and concentration-driven motility. Fig 2 shows the growth curves of the three progression patterns generated for the treated (Fig 2a, 2c and 2e) and the curves of the untreated (Fig 2b, 2d and 2f) tumors. The corresponding simulated magnetic resonance imaging are shown in Fig 3a, 3c and 3e for treated tumors and Fig 4a, 4c and 4e for untreated ones.

**Table 4:** Hypoxia-driven versus concentration-driven motility: simulated progression patterns.

Motility	High Hypoxia-Driven (1.4 × 10^−3^ *mm*/*hr*)	Low Hypoxia-Driven (1.4 × 10^−4^ *mm*/*hr*)
**High Concentration-Driven** (4 × 10^−3^ *mm*^2^/*hr*)	Expanding FLAIR	Expanding FLAIR
**Medium Concentration-Driven** (8 × 10^−5^ *mm*^2^/*hr*)	Expanding FLAIR + Necrosis	Expanding FLAIR + Necrosis
**Low Concentration-Driven** (8 × 10^−7^ *mm*^2^/*hr*)	Expanding Necrosis (++)	Expanding Necrosis (+)

Tumor progression for the three progression patterns can occur by an increase in either FLAIR, necrosis, or both. The model generates the three progression types with different titrations of the hypoxia-driven and concentration-driven motility rates. In the table, plus signs (+) indicate the relative size of expanding necrosis in the simulations for low values of the concentration-driven motility rate.

The results of our simulations reveal that highly-dispersive tumors generate the progression pattern of Expanding FLAIR without new necrosis beyond the original site of the tumor (Fig 3). Treatment effectively reduces the high density proliferating tumor mass. After anti-angiogenesis therapy, partial tumor and brain death occurs initially at the site of the tumor; however, necrosis never exceeds 1.5% of the total brain (green arrows), compared to an original high density tumor mass encompassing 2.4% of the brain (red arrows). Following treatment, tumor cells continue to spread throughout the brain albeit at much lower densities (white arrows), which could explain why the magnetic resonance imaging appearance at progression for this progression pattern shows an increase in the appearance of FLAIR but no new visible gadolinium enhancement. The simulation in Fig 3a shows 84% FLAIR at patient death. The growth curves in Fig 2 plot the percent growth of FLAIR, necrosis, and high density tumor mass for treated and untreated tumors in the brain. Note that for the highly-dispersive tumor (Fig 2a and 2b), the necrosis curve is constant (*i.e*. no increase) in the treatment model while the FLAIR curve continues to increase.

For a moderate concentration-driven motility parameter (Table 4, moderate), we observe glioblastoma multiforme progression by Expanding FLAIR + Expanding Necrosis, which is the new progression pattern identified in [6]. Fig 3c and 3d display this progression pattern. When this moderately-dispersive tumor is treated (first columns), there is a decrease in the proliferating tumor mass, or gadolinium enhancement, but necrosis and FLAIR continue to expand, as evidenced by the growing hole in the brain (green arrows) and the presence of brain invasion (white arrows) beyond the site of necrosis. In contrast to the simulations with highly-dispersive tumor, the area of necrosis at patient death (4.2% for Fig 3c and 5.0% for Fig 3d) exceeds the area of the original tumor mass (2.6% for both). The area of FLAIR at patient death in Fig 3c covers 33% of the brain. Notice that in Fig 2c, necrosis and FLAIR both continue to expand in the moderately-dispersive tumor, with the difference between the two areas increasing in time.

In the presence of very low concentration-driven motility (Table 4, low), the high parameter choice for hypoxia-driven motility generates a glioblastoma multiforme tumor that progresses by Expanding Necrosis. For this reason, we associate this progression pattern with a hypoxia-driven tumor, defined as having low concentration-driven and high hypoxia-driven motility. The high hypoxia-driven motility parameter value results in an aggressively expanding area of necrosis, indicated by (++) in Table 4 and shown in Fig 3e. Note that in the hypoxia-driven tumor simulation (Fig 3e), invasive cells remain in close proximity to the site of necrosis, which is consistent with the magnetic resonance imaging indicators distinguishing Expanding Necrosis from Expanding FLAIR + Necrosis. In this case, the area of necrosis (green arrow) at patient death covers 4.2% of the brain with a comparable area of FLAIR (white arrow) covering 6% of the brain. The plot in Fig 2e best illustrates this relationship between FLAIR and necrosis: the growth curves for each increase at the same rate in time following the initiation of anti-angiogenesis therapy. It is noteworthy that the combination of low concentration-driven and low hypoxia-driven motility parameter values produces a comparatively benign tumor, which is not consistent with the aggressive behavior of glioblastoma multiforme (Table 4 and Fig 3f).

### Motility Phenotypes Determine Survival Times in Anti-angiogenesis Therapy

The section above illustrates that the motility phenotypes predict the progression patterns in glioblastoma multiforme patients treated by anti-angiogenesis. The second goal of this investigation was to test the hypothesis that the motility phenotypes (*i.e*. concentration-driven and hypoxia-driven motility) not only control the progression patterns identified in [6] and [5] but also the associated overall survival previously discussed. Hence, we simulate a clinical trial by applying treatment to each of the three tumor types (highly-dispersive, moderately-dispersive, hypoxia-driven) and by measuring the overall survival following the initiation of treatment (time of death—time of treatment). We also include a control group in the experimental design that consists of simulated untreated tumors from each of the three tumor groups. Table 5 summarizes the experimental design of the simulated trial as well as the median overall survival time found for each group.

**Table 5:** Experimental design of the simulated clinical trial.

Trial Group	Group Size	Motility Phenotype	Treatment Criteria	Death Criteria	Median Survival Time
**Highly-Dispersive + Anti-Angiogenesis**	30	Concentration-Driven: *High* Hypoxia-Driven: *High*	Tumor: 2.0–2.8%	FLAIR: 55–88%	16.3 months
**Moderately-Dispersive + Anti-Angiogenesis**	25	Concentration-Driven: *Moderate* Hypoxia-Driven: *High*	Tumor: 2.0–2.8%	Necrosis: 3.5–4.4%	2.0 months
**Hypoxia-Driven + Anti-Angiogenesis**	25	Concentration-Driven: *Low* Hypoxia-Driven: *High*	Necrosis: .09–.32%	Necrosis: 3.5–4.4%	2.3 months
**Control (Not Treated)**	25	Concentration-Driven: *High* Hypoxia-Driven: *High*	Tumor: 2.0–2.8%	Tumor: 10–18%	2.5 months
	25	Concentration-Driven: *Moderate* Hypoxia-Driven: *High*	Tumor: 2.0–2.8%	Necrosis: 3.5–4.3%	
	25	Concentration-Driven: *Low* Hypoxia-Driven: *High*	Necrosis: .09–.32%	Necrosis: 3.5–4.4%	

Results of the 30 simulations in the anti-angiogenesis-treated highly-dispersive tumor group revealed a median overall survival time of 16.3 months (Table 5), and an average overall survival time of 16.0 months, which is comparable to the median overall survival time of 17.7 months reported in [5] for this progression pattern. For the anti-angiogenesis-treated moderately-dispersive tumor group, the results of 25 simulations revealed an average post-treatment overall survival time of 5 months with a median overall survival time of 2 months. This short overall survival time is consistent with the poor prognosis associate with this progression pattern reported in [6]. Finally, for the hypoxia-driven tumor, the results of 25 simulations revealed an average post anti-angiogenesis overall survival of 2.3 months with a median overall survival time of 2.3 months. Nowosielski *et al*. reported a similarly low median overall survival time of 5.0 months for this particular progression pattern [5].

In order to provide a basis for comparison to the primary non-responder group discussed in [5], we ran 25 “controls”, or untreated, for each of the three tumor types for a total of 75 simulations. Among the untreated tumor group, there was an average overall survival time of 3 months following diagnosis with a median overall survival time of 2.5 months. Recall that the average overall survival time among the treated hypoxia-driven tumors was similarly low and that Nowosielski *et al*. also reported comparable low average overall survival time between this progression pattern (5.0 months) and primary nonresponders (4.9 months) [5].

**Fig 5:**
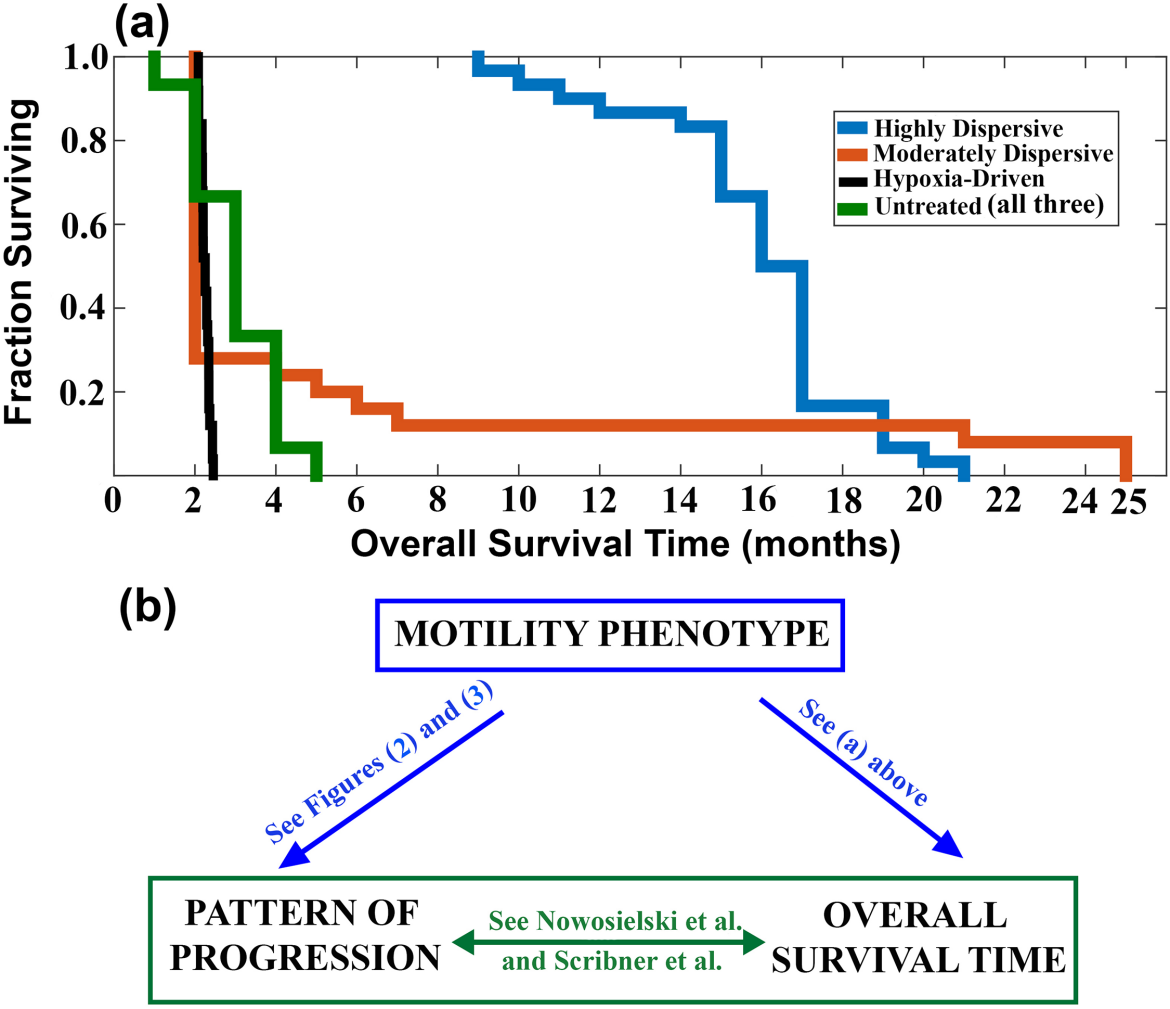
Predicting patients response and survival times. (a) is a Kaplan-Meier analysis of the overall survival of the four tumor groups in the computational trial. There are three treatment groups: 30 highly-dispersive Tumors (blue), 25 moderately-dispersive (red), and 25 hypoxia-driven Tumors (Black). The control group (green) includes 75 untreated tumors from all three tumor groups. The difference in overall survival between the highly-dispersive tumors and all other groups is significant (Log-Rank *p* < 0.01); the differences between moderately-dispersive and both the hypoxia-driven and untreated groups is not significant. (b) is a cartoon that illustrates how the motility phenotypes can predict both the patterns of progression and the overall survival times (blue arrows).

Fig 5a displays the results of a Kaplan-Meier analysis of the three treated tumor types and the untreated tumors. For our computational trial, we found Log-Rank *p* = 0.0087 for highly-dispersive vs moderately-dispersive; the differences between moderately-dispersive and both the hypoxia-driven and untreated groups (controls) was insignificant; for all other comparisons, Log-Rank *p* < 0.001. Nowosielski *et al*. similarly found significant differences in the post-treatment overall survival (Log-Rank *p* = 0.001) of the highly-dispersive tumor (T2-diffuse or progression by FLAIR), the hypoxia-driven tumor (T2-Circumscribed or progression by Necrosis), and the primary nonresponders.

Figs 3 and 4 show that in the presence of low concentration-driven motility, varying the parameter choice for hypoxia-driven motility exerts a significant effect on the rate of tumor growth in both the treated and untreated models. Notice the significant difference in overall survival times of 3 months (high hypoxia-driven motility parameter) verses 33 months (low hypoxia-driven motility parameter) in the tumors treated by anti-angiogenesis (Fig 3e and 3f). The difference in overall survival times is also notable (3 months verses 9 months) in the untreated tumor.

### Efficacy of Rate- And Motility-Reducing Agents in Each Phenotype

**Fig 6:**
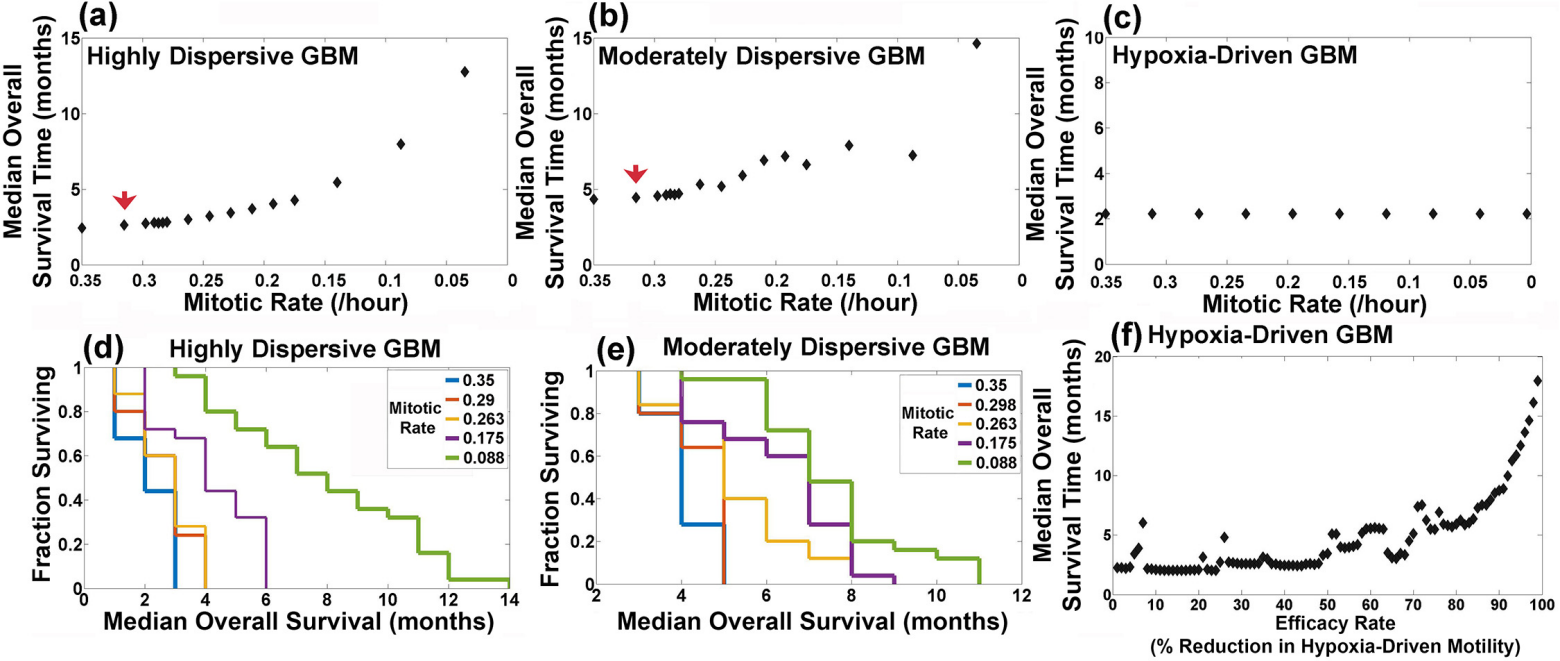
Effects of replication rates on overall survival time. (a)—(c) plot the results of computational trials studying the effects of titrating the motility rate (/hr) on the overall survival of patients with highly-dispersive, moderately-dispersive, and hypoxia-driven glioblastoma multiforme (GBM), respectively. Each dot corresponds to the median overall survival of a group of 25 patients (Log-Rank *p* < 0.05). The red arrows in (a) and (b) indicate the highest mitotic rates that generate statistically significant prolongation of overall survival as compared to baseline rate of 0.35/hr. (d) and (e) show selected Kaplan-Meier survival curves from the computational trials (a) and (b), respectively. (f) plots the relationship between the efficacy of a motility-reducing agent and overall survival times in patients with hypoxia-driven GBM; each dot represents the median overall survival time of a group of 25 patients.

The section above illustrates that the motility phenotypes predict both the progression patterns and overall survival times of glioblastoma multiforme patients treated by anti-angiogenesis (see Fig 5). In this section, we investigate how each phenotype responds to rate-reducing agents, like Tumor Treating Fields. We design computational trials by titrating the replication rate in each of the three phenotypes and assign 25 patients to each replication rate. Fig 6a–6c plot the median overall survival time associated with all the titrations; selected Kaplan-Meier curves are shown in Fig 6d and 6e. The results reveal that rate-reducing agents may prolong the overall survival times of patients with highly-dispersive and moderately-dispersive glioblastoma multiforme (Fig 6a and 6b) but not patients with hypoxia-driven glioblastoma multiforme (Fig 6c). In highly-dispersive and moderately-dispersive tumors, statistically significant prolongation of overall survival times is observed when the replication rates are below 0.29/hr and 0.298/hr, respectively (Log-Rank *p* < 0.05). It is noteworthy that the growth of hypoxia-driven glioblastoma multiforme appears to be driven mainly by motility as significant reductions in replication rates exert no effects on overall survival (Fig 6c). Because hypoxia-driven glioblastoma multiforme exhibits no response to severe reductions in replication rates (see Fig 6c), we evaluate the effects of motility-reducing agents by the titrating hypoxia-driven motility parameter. The findings corroborate the observation that motility-reducing agents prolong the overall survival of patients with hypoxia-driven glioblastoma multiforme (Fig 6f).

### Relationship Between Efficacy and Overall Survival Times in a Computational Population Trial

Current glioblastoma multiforme clinical trials do not include stratification by motility phenotype nor by the rate of replication of the tumor. A computational population trial can be used to describe such a design. With such trials we evaluate the potential clinical benefits of rate-reducing agents by looking for a relationship between efficacy and overall survival. Efficacy of a rate-reducing (motility-reducing) agent is defined as the percent lowering of the mitotic (motility) rate.

**Fig 7:**
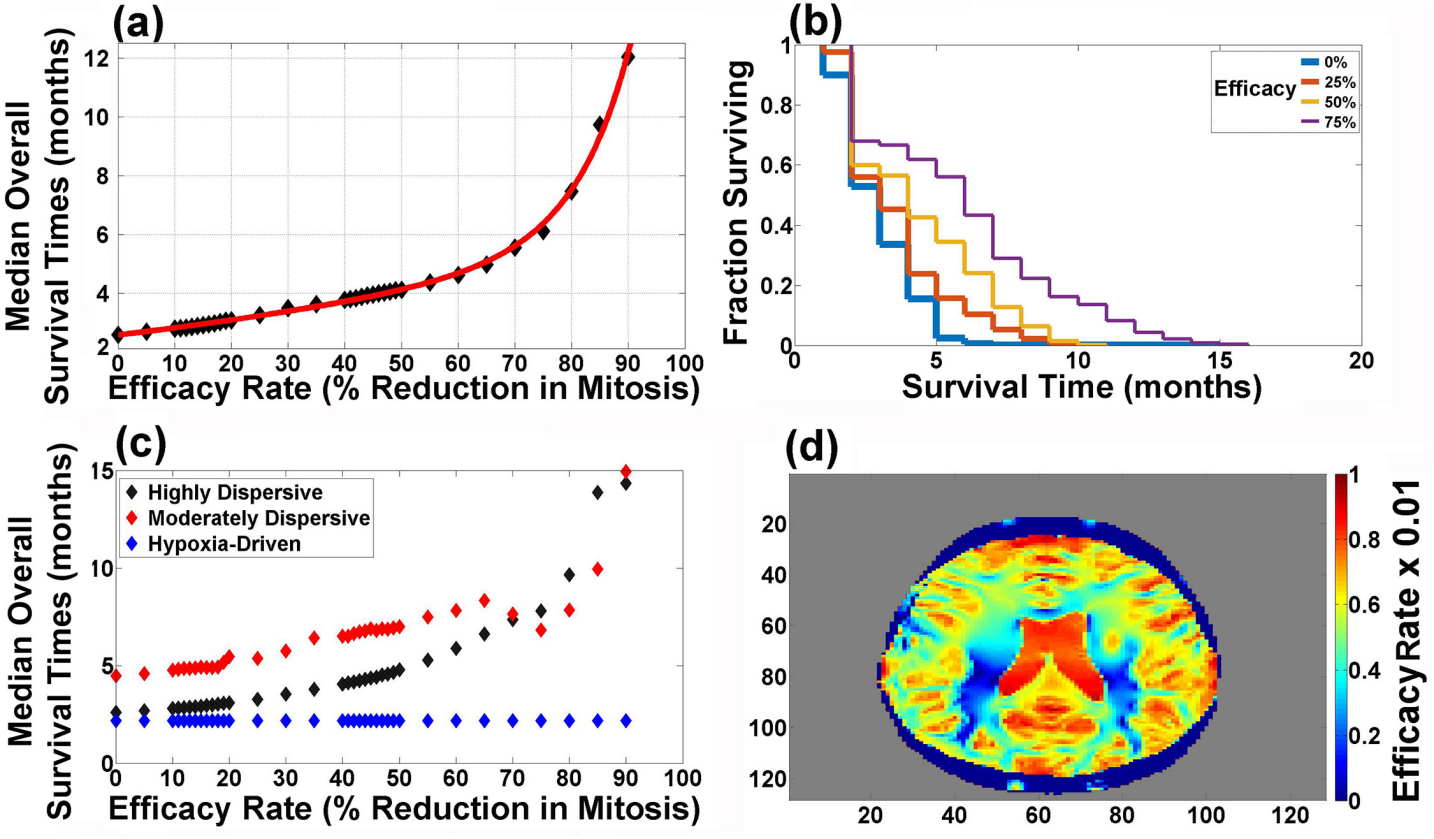
Population trial: effects of replication and motility rates on overall survival time. (a) plots the results of the population computational trial, that includes all three glioblastoma multiforme phenotypes and different mitotic rates. Each point corresponds to the median overall survival time of a group of 975 patients. The data fits into a bi-exponential model (red line), *f*(*x*) = *ae*^*bx*^ + *ce*^*dx*^. The coefficients (with 95% confidence bounds) are: *a* = 2.584 (2.51, 2.657), *b* = 0.00889 (0.008012, 0.009768), *c* = 0.0005212 (1.322×10^−5^, 0.001029), *d* = 0.1047 (0.09417, 0.1152). Goodness of fit: SSE: 0.3315, R-square: 0.9975, Adjusted R-square: 0.9973, RMSE: 0.1034. (b) shows selected Kaplan-Meier survival curves from the population trial including all three glioblastoma multiforme phenotypes shown in (a). (c) plots the effects of the efficacy of a rate-reducing agent in each of the three glioblastoma multiforme phenotypes. (d) plots the computed factor of reduction in tumor mitotic rates generated by Tumor Treating Fields (*i.e*. efficacy rate ×0.01) in response to a spatial distribution of the electric field in the brain.

We designed such a population trial for investigating the efficacy of Tumor Treating Fields, which produce a spatial gradient of reduction of the mitotic rate. Note that this is in contrast to the simulations performed in Fig 6, where the percent lowering of mitotic rate was uniform and everywhere the same. Each efficacy rate (reduction by 0–90%) is evaluated by a computational population trial including all three motility phenotypes as well as tumors with variable replication rates. The three phenotypes (highly-dispersive, moderately-dispersive, hypoxia-driven) are represented equally in each trial, based on their reported clinical incidence in glioblastoma multiforme patients [5, 6]. Furthermore, thirteen different initial mitotic rates are chosen randomly within the interval [0.35/hour, 0.294/hour]; the lower limit corresponds to the average of the two upper bounds that generate statistically-significant prolongation of overall survival times in both highly-dispersive and moderately-dispersive glioblastoma multiforme (red arrows in Fig 6a and 6b). Twenty-five patients are assigned to each replication rate. Each point in Fig 7a depicts the median overall survival time of 975 patients (25 (patients) x 3 (glioblastoma multiforme phenotypes) x 13 (replication initial rates); the x-axis corresponds to the efficacy of the agent (i.e. percent reduction in replication rate). Selected Kaplan-Meier survival curves of the population computational trial including all three phenotypes are shown in Fig 7b.

The results yield a relationship between the efficacy of the rate-reducing agent and overall survival times; in particular, an efficacy rate of 75% causes an increase of the median overall survival time from 2.5 to 6 months (Fig 7a). Fig 7c plots the relationships between efficacy and the overall survival time in each of the three glioblastoma multiforme phenotypes. Fig 7d depicts the computed factor of reduction in tumor mitotic rates generated by Tumor Treating Fields (i.e. Efficacy of Tumor Treating Fields ×0.01) in response to the spatial distribution of the electric field in the brain. These simulations reveal that Tumor Treating Fields generate efficacy rates in targeted spatial areas of the brain that could potentially yield a clinical response leading to prolongation of overall survival times in glioblastoma multiforme patients (Fig 7d). It is also noteworthy that the spatial distribution of the efficacy rates in the brain can be modified by the placement of the arrays [16]. Furthermore, excluding the hypoxia-driven tumors could potentially enhance the clinical response (Fig 7c).

## Discussion

**Fig 8:**
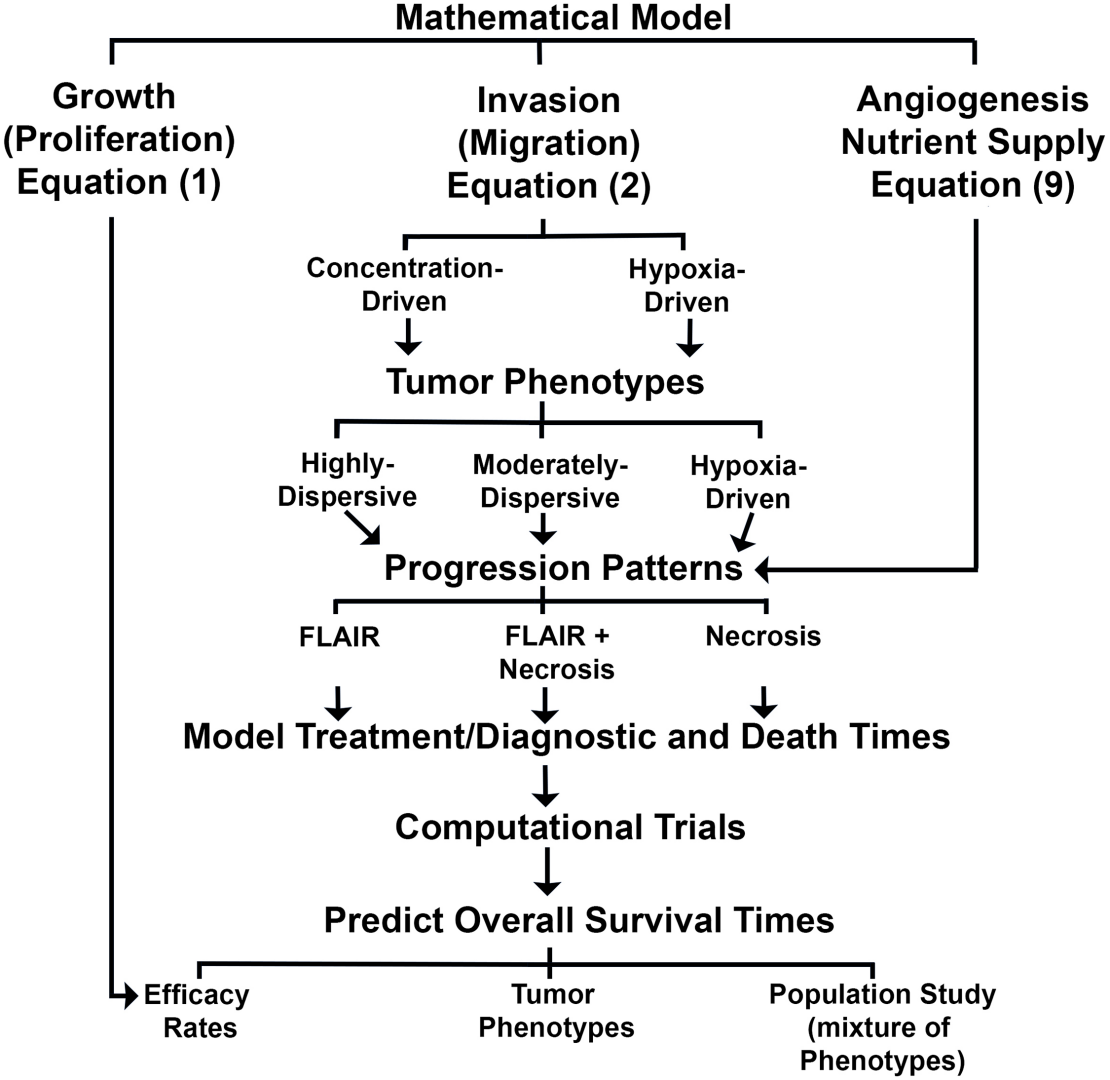
A diagram of the components of the model, tumor phenotypes, progression patterns, and computational trials to predict overall survival times.

Here, we apply a mathematical model to identify motility phenotypes that replicate the progression patterns of glioblastoma multiforme and conduct computational trials whose results reproduce the reported clinical overall survival times (see flow chart in Fig 8). In particular, we conduct computational trials to study the therapeutic effectiveness of mitotic rate- and motility-reducing agents (see Table 6). The results support the idea that mitotic-rate reducing agents are effective in highly-dispersive and moderately-dispersive glioblastoma multiforme while motility-reducing strategies are effective in hypoxia-driven tumors. A population computational trial, including all three phenotypes, reveals a relationship between the efficacy of the mitotic-reducing agent and overall survival times; for example an efficacy of 75% prolongs the overall survival by 3.5 months (Fig 7a). Considering the invasiveness of glioblastoma multiforme and the inhomogeneous spatial distribution of the efficacy rates of Tumor Treating Fields (Fig 7d), these results are consistent with the reported prolongation of overall survival for patients treated in the Tumor Treating Fields Phase 3 clinical trial (4 months) [4]. Nonetheless, it is also possible that Tumor Treating Fields may yield long overall survival time of a small moderately-dispersive glioblastoma multiforme treated by efficacy rates exceeding 90% (see Fig 6b) [4, 17].

**Table 6:** Summary of the phenotypes of glioblastoma multiforme and their predicted response to anti-angiogenesis, anti-mitotic, and anti-hypoxia driven motility agents.

Tumor Phenotype	Response to Anti-Angiogenesis	Response to Anti-Mitotic Agents	Response to Anti-Hypoxia Driven Motility Agents
**Highly-Dispersive**	Survival: ++ PP: Expanding FLAIR	Survival: Increases with Efficacy	Survival: Minimal or no Response
**Moderately-Dispersive**	Survival: + PP: Expanding FLAIR + Necrosis	Survival: Increases with Efficacy	Survival: Minimal or no Response
**Hypoxia-Driven**	Survival: No response PP: Expanding Necrosis	Survival: No Response	Survival: Increases with Efficacy

+ and ++ indicate short and long prolongation of survival times, respectively. PP refers to progression pattern.

Eikenberry *et al*. reported a model of glioblastoma multiforme growth and invasion [15]. They used a three-dimensional brain geometry and simulated surgical resection, radiotherapy and chemotherapy. Our model also generates three-dimensional simulations that are consistent with the aforementioned results. Eikenberry *et al*. found survival benefits with large tumor resections. Although, the clinical questions related to the use of anti-angiogenesis addressed in this paper do not typically include a surgical resection, our computational trials reveal that the overall survival times are negatively correlated with the tumor mass at the start of treatment with anti-angiogenesis (data not shown). Hathout *et al*. introduce a model that includes an advection term to account for the so-called cell streaming, where some of the tumor cells seen to stream widely along white matter pathways [18]. Other models attempt to capture the dynamics of glioblastoma multiforme by passive diffusion only [19–22]. Swanson *et al*. has introduced the proliferation-invasion-hypoxia-necrosis-angiogenesis model (PIHNA) that stipulates the presence of 2 glioma cell populations in normoxic and hypoxic states [23]. A complex model, developed by Frieboes *et al*. [24], includes mechanical stress, interactions with extra-cellular matrix, angiogenesis, and growth-promoting factors. Neither the models of Frieboes or Swanson consider the hypoxia-driven or the go-or-grow phenotypes.

Our model enhances oxygen/nutrient supply by allowing a higher density of tumor cells. Anti-angiogenesis therapy abrogates this capacity. Although, these ideas are consistent with the mechanisms of angiogenesis and anti-angiogenesis, we are not making any assumptions on vessel regression or normalization in response to anti-angiogenesis therapy. Growing glioblastoma multiforme tumors produce vascular endothelial growth factor to promote angiogenesis, which provides a conduit for blood flow to deliver nutrients and oxygen in order to meet the metabolic demands of the growing neoplasm. In turn, as the tumor enlarges, necrotic and hypoxic zones are created in tumors due to the immature nature of tumor vasculature, especially if the speed of tumor replication exceeds the rate of angiogenesis [25]. Therefore, in certain regions of tumors, the vessels may be highly permeable and ectatic. These immature vessels do not provide nutritive flow thus contributing to the hypoxic environment. The Jain vascular normalization hypothesis stipulates that anti-angiogenic therapy could induce a transient normalization of the structure and function of some blood vessels in the tumor by improving vascular permeability, organization, and perfusion [26]. During this normalization window, which lasts from hours to days after vascular endothelial growth factor blockade, there is improvement in tumor oxygenation, drug delivery, and radiation sensitivity. In the case of glioblastoma multiforme, it appears that tumor cell-derived angiopoietin-1 is an absolute requirement for normalization [27]. The same model used in this investigation was applied to simulate vascular normalization; the results are consistent with the Jain hypothesis in showing a transient increase in tumor growth [6]. Vascular endothelial growth factor is a potent mediator of vascular permeability and blood brain barrier disruption in brain tumors [28, 29]. Thus, bevacizumab has been postulated to exert steroid-like actions in glioblastoma multiforme [30]. However, these steroid-like effects do not explain all the biological effects/actions of bevacizumab because steroids do not cause: 1) the three progression patterns, studied in this paper (Figs 2 and 3) and 2) a median overall survival time of 17.7 months in patients with the progression pattern of Expanding FLAIR (see Fig 5 and [5]).

The research suggests new insights and hypotheses on the basic biology of glioblastoma multiforme:

Motility as a Biomarker. The results link the mechanisms and rate of motility to the progression patterns and overall survival times of patients diagnosed with glioblastoma multiforme. Identifying a subgroup of glioblastoma multiforme that could potentially respond to anti-angiogenesis is desirable. For example, our findings suggest the hypothesis that bevacizumab is expected to prolong the overall survival time of a patient with a highly-dispersive tumor but it is futile in moderately-dispersive and hypoxia-driven tumors. Similarly, a mitotic rate-reducing agent, like Tumor Treating Fields, is expected to be beneficial in highly-dispersive and moderately-dispersive tumors but futile in hypoxia-driven tumors, unless Tumor Treating Fields are found to reduce motility as well. The remarkable results on the behavior of the aggressive and resistant hypoxia-driven glioblastoma multiforme highlight the need to develop motility-reducing therapeutic agents.
“Motility Principle”. In the treated tumors, adjustments in hypoxia-driven motility from high to low have minimal or no effects on tumor progression in the presence of high or moderate concentration-driven motility (see Fig 3a–3d). However, the effects of hypoxia-driven motility surface when the rate of concentration-driven motility is low (See Fig 3e and 3f). The observation applies to the untreated tumor as well (See Fig 4). Then, high hypoxia-driven motility produces a much more aggressive untreated tumor as compared to low hypoxia-driven motility, as seen in Fig 4e and 4f. This observation led us to the “Motility Principle”, which states that hypoxia-driven motility has no significant bearing on the behavior of the tumor unless concentration-driven motility is very low. The mathematical terms governing these two types of motility offer insights into the mechanisms behind this principle (Fig 1).
Hypoxia-driven Motility Couples Invasion to Necrosis. The distinguishing feature of hypoxia-driven motility is that it only permits invasive cells to travel up the concentration gradient of brain tissue, causing the cells to accumulate sharply at the hypoxic edge of the tumor (see Fig 1b). This spike in tumor cell concentration accelerates the onset of necrosis, which in turn produces a gradient of brain tissue for the invasive cells to move along. Hypoxia-driven tumors are characterized by a positive feedback loop of invasion and necrosis, where invasive cells are followed closely by necrosis (Fig 1a and 1b).
Concentration-driven Motility: Uncoupling Invasion and Necrosis. Clinical and computational data demonstrate that highly-dispersive tumors treated by anti-angiogenesis have relatively long overall survival times (Fig 5). Our results predict that patients with untreated highly-dispersive glioblastoma multiforme have a shorter overall survival times than those with moderately-dispersive tumors (Fig 5c). One possible mechanistic explanation for this paradoxical behavior is that necrosis and high density tumor govern the overall survival in the case of the untreated tumors. However, in the presence of anti-angiogenesis, the time of death of patients with highly-dispersive tumors is controlled by the percent of brain invaded by tumor cells (FLAIR), which can slowly cover a larger percentage of the brain (as compared to necrosis and high density tumor) before killing the patient (Table 5).
Future Studies. Determining the motility phenotype of a glioblastoma multiforme is desirable as it appears to exert a significant impact on therapeutic choice and efficacy and on survival times. The development of methodology for calibration (*i.e*. parameter estimation) using magnetic resonance images holds the potential of generating a classification of glioblastoma multiforme based on its motility phenotype (*i.e*. highly-dispersive, moderately-dispersive, and hypoxia-driven) and its replication rate; this classification can optimize therapeutic choice, the spatial configuration of Tumor Treating Fields, and can predict the time of and pattern of recurrence.
  The rate of failure of phase 3 clinical trials is remarkably high. Estimates by the Food and Drug Administration of the success rates of biopharmaceuticals that entered clinical trials in oncology throughout the 90’s, is estimated at approximately 8% (FDA Challenges and Opportunities Report, March 2004) [31, 32]. The success rate is even lower for neurological applications, like neuroprotection in ischemic stroke [33, 34]. The high failure rates of expensive phase 3 trials elevate the cost of drug development. This research demonstrates the potential of computational trials in testing hypotheses, validating phenotypes, and predicting clinical data. Computational trials, built on the basis of mechanisms of actions and therapeutic efficacy, have the potential of improving the success rates of phase 3 clinical trials, thus lowering the cost of drug development and optimizing target discovery.

## Materials and Methods

### Equations

The authors have recently reported a system of partial differential equations that models glioblastoma multiforme biology at the scale of magnetic resonance imaging; the model includes replication, brain invasion, angiogenesis, and hypoxia [6, 35]. Here, we use the system reported and described in [6]. The equations are shown below, and the parameter values and units may be found in Table 7. There are only two modifications from the system presented in [6]. First, the degradation term in each equation has been altered to accommodate a maximum threshold for the tumor mass and to ensure that no cell survives in the necrotic core. We multiplied the previously used degradation rate in [6] by $\frac{(P+I)/100+1}{B+0.01}$. Second, the equation measuring the level of local hypoxia in the brain is now a continuously differentiable function, as opposed to a piece-wise continuous function, which is preferred when solving systems of equations numerically. The equations and parameter choices are shown below. A parameter sensitivity analysis was performed in [6].

**Table 7:** Description of the Parameters and Values.

Description	Symbol	Value Used
Transition rate from *P* to *I*	*α*	2.02/*hr*
Transition rate from *I* to *P*	*β*	2.00/*hr*
Mitotic rate (max) of *P* cells	*τ*	0.35/hr
Diffusion coefficient of *I* cells	*δ*	[8 × 10^−7^,4 × 10^−3^]*mm*^2^/*hr*
Diffusion Tensor in Brain	*D*	1.0
Necrotic rate of living cells	*γ*	0.17/*hr*
Active transport of *I* cells	*η*	[1.4 × 10^−4^,1.4 × 10^−3^]*mm*/*hr*
Initial Hypoxic threshold	Ω	1.1
Angiogenic rate	*σ*	1.5
Fixed difference: *C*_ltm_−*C*_hyp_	Φ	0.10

In the equation describing invasive cell movement, originally published in [6] and reprinted below, there are two distinct terms governing these two types of motility. The first migration term, *δ*∇ ⋅ (*D*∇*I*), describes concentration-driven motility. It is a classic diffusive term, which means that the rate of movement of the invasive cells is proportional to its own concentration gradient and independent of other factors like local hypoxia. Another parameter, D, varies spatially to replicate the increased rate of movement of cancer cells along blood vessels or white matter tracks in the brain. Ultimately, the values for D will depend on the individual topography of the brain, allowing for more patient-specific tumor simulations. Combined, this passive diffusion term translates into increased tumoral movement in all directions away from the areas of highest invasive cell concentrations, which usually occurs on the boundary of tumor. In contrast to hypoxia-driven, concentration-driven motility is unresponsive to oxygen availability, with the exception that it will not allow invasive cells to diffuse into the necrotic core. White matter tracks (bundles of axons) and blood vessels are assumed dead (D ≈ 0) at the necrotic core of the tumor (defined by 80% or more brain death). Thus invasive cells will not diffuse back into the dead center of the tumor.

The second migration term, *η*∇ ⋅ (*I*∇*B*), governs hypoxia-driven motility and reflects the preferential movement of invasive cells in the direction of healthy brain tissue and away from necrosis. Moreover, the speed of migration is proportional to both the concentration of invasive cells and the gradient of normal brain tissue, ∇*B*. As illustrated in Fig 1b, invasive cells located on the hypoxic edge of the tumor where the concentration of brain cells steeply increases in one direction will move away from the tumor core by hypoxia-driven motility in search of more nutrients. Likewise, as invasive cells approach healthy regions of the brain where the concentration gradient of normal brain tissue is close to 0, speed of movement in that direction will gradually decrease to zero. Hence, an important distinction between the two motility types is their movement in healthy brain tissue (Table 3). Because the driving force for hypoxia-driven motility in our mathematical model is the gradient of brain tissue, invasive cells driven by hypoxia-driven motility alone will not move throughout healthy brain tissue. This difference is illustrated in Fig 1b by the accumulation of red invasive cells at the edge of healthy brain tissue in the cartoon for hypoxia-driven motility (see double-sided red arrow labeled FLAIR). The time evolution curves also illustrate this difference, with invasive cells sloping gradually into healthy brain in the concentration-driven motility model and accumulating with a sudden and sharp decrease at the edge of healthy brain in the hypoxia-driven motility model.

The system of equations is

$$\text{Proliferative Cells}:\partial_{t}P\;=\;\underset{\begin{array}{c} \text{Net production of }\\ \text{P cells} \end{array}}{\underset{︸}{MP}}-\underset{\begin{array}{c} \text{Conversion of}\\ \text{P cells to I}\\ \text{during hypoxia} \end{array}}{\underset{︸}{\alpha HP}}+\underset{\begin{array}{c} \text{Conversion of}\\ \text{I cells to P}\\ \text{during normoxia} \end{array}}{\underset{︸}{\beta \left(1-H\right)I}}-\underset{\begin{array}{c} \text{Necrosis of}\\ \text{P cells}\\ \text{during hypoxia} \end{array}}{\underset{︸}{\gamma FP}}$$ (1)

$$\text{Invasive Cells}:\partial_{t}I\;=\;\underset{\begin{array}{c} \text{Passive diffusion }\\ \text{of I cells} \end{array}}{\underset{︸}{\delta \nabla \cdot \left(D\nabla I\right)}}-\underset{\begin{array}{c} \text{Active transport}\\ \text{of I cells} \end{array}}{\underset{︸}{\eta \nabla \cdot \left(I\nabla B\right)}}+\underset{\begin{array}{c} \text{Conversion of}\\ \text{P cells to I}\\ \text{during hypoxia} \end{array}}{\underset{︸}{\alpha HP}}-\underset{\begin{array}{c} \text{Conversion of}\\ \text{I cells to P}\\ \text{during normoxia} \end{array}}{\underset{︸}{\beta \left(1-H\right)I}}-\underset{\begin{array}{c} \text{Necrosis of}\\ \text{I cells}\\ \text{during hypoxia} \end{array}}{\underset{︸}{\gamma FI}}$$ (2)

$$\text{Brain Cells}:\partial_{t}B=\underset{\begin{array}{c} \text{Necrosis of}\\ \text{brain cells}\\ \text{during hypoxia} \end{array}}{\underset{︸}{-\gamma FB}}$$ (3)

$$\text{Necrotic Cells}:\partial_{t}N\;=\;\underset{\begin{array}{c} \text{Conversion of P, I, and B}\\ \text{to necrotic cells}\\ \text{during hypoxia} \end{array}}{\underset{︸}{-\gamma F(B+I+P)}}$$ (4)

Assumptions regarding angiogenesis, hypoxia, mitosis, and necrosis:

Total cell concentration:C=P+I+B+N (5)

$$\text{Measure of Local Hypoxia}:H=\left(\frac{1-\tanh(40\left(C_{hyp}-C\right))}{2}\right)$$ (6)

Mitotic Rate:M(H)=τ(1-H) (7)

$$\text{Rate of necrosis}:\gamma F=\gamma \left(\frac{\frac{P+I}{100}+1}{B+0.01}\right)\left(\frac{1-\tanh(40\left(C_{ltm}-C\right))}{2}\right)$$ (8)

$$\text{Hypoxic threshold}:C_{hyp}=\sigma \left[\text{log}\left(1+P\right)\right]+\Omega ,$$ (9)

where *σ* = 1.5 to simulate angiogenesis, and *σ* = 0 to simulate anti-angiogenesis.

$$\text{Necrotic threshold}:C_{ltm}=C_{hyp}+\Phi$$ (10)

A description of the parameters and their values/units may be found in Table 7.

### Numerical Methods

Simulations were obtained using finite difference schemes in a program written in C. The brain was discretized into a 112 x 83 spatial mesh with each voxel measuring approximately 2.25 *mm*^2^ in area, and a small concentration of both proliferative cells (on the order of 10^−1^) and invasive cells (on the order of 10^−2^) was inserted into a single voxel at the start of the simulation. The initial topography of the brain was taken from a virtual magnetic resonance imaging slice and included brain matter as well as skin and bones, which are assumed impermeable to the migrating tumor (http://brainweb.bic.mni.mcgill.ca/brainweb/). The computational trials were run on 200 processors of the Alabama Supercomputer Authority (Huntsville, AL); they were completed in nearly two hours.

### Analysis of Simulated Magnetic Resonance Imaging

Fig 3 displays the results of six different glioblastoma multiforme tumor simulations using our system of equations. For the purpose of this investigation, an area of the brain is considered necrotic if more than 80% of the brain cells are dead in a given cell (green arrows), and the presence of a high-density tumor is detected if the sum of proliferative and invasive cells exceeds a concentration of 0.7 in a given voxel (red arrows). Likewise, FLAIR, is detected when the sum of proliferative and invasive cell concentrations falls between 0.05 and 0.7 (white arrows).

### Simulated Clinical Trials

In this investigation, we simulate a clinical trial by applying treatment, monitoring tumor growth, FLAIR and necrosis, and by measuring the overall survival time following the start of treatment. This overall survival time is taken to be the difference in the time of death and the time of treatment. We define the time of treatment to be the time in the simulation when either the size of the high density tumor or the area of brain necrosis reaches a certain critical threshold. Since the tumor size and necrosis differ among patients, we begin treatment when the first of either two criteria (high density tumor size or amount of necrosis) is met. Anti-angiogenesis treatment is simulated by setting the angiogenic rate of the tumor equal to zero (see Table 7). Likewise, the time of death is defined as the time when either the area of FLAIR or necrosis in the brain reaches a critical size. As in the time of treatment, the time of death is triggered when the first of these two thresholds is reached (Table 5). We also include a control group in the experimental design that consists of simulated untreated tumors from each of the three tumor groups. The overall survival for this group is taken to be the difference in the time of death and the time of diagnosis. We define the time of diagnosis the same way as the time of treatment, only there is no change in the angiogenic rate of the tumor.

Symptoms leading to glioblastoma multiforme diagnosis can vary from patient to patient depending on many factors, like the location of the tumor in the brain or the size of the tumor. Similarly, the use of steroids and other factors may also cause variations in the time of death among patients. Hence, in order to obtain 25–30 simulations for each of the three treatment groups, we titrate the criteria needed to initiate tumor treatment as well as the criteria needed to determine the time of death. To titrate treatment, we vary the area of the high density tumor needed to initiate therapy between 2% and 2.8% of the total brain, and we vary the amount of brain necrosis needed to initiate treatment between 0.09% and 0.32% of the brain. To titrate time of death, we also use one of two criteria–percent of brain invaded (*i.e*. FLAIR) or percent of brain that is necrotic. The amount of FLAIR needed to determine time of death varies between 55% and 88% of the total brain, and the amount of brain necrosis varies between 3.5% and 4.4% of the brain.

For the control group (untreated), we perform 25 simulations for each of the three tumor types (for a total of 75 simulations) and vary the diagnostic time as well as the time of death. Criteria used to vary the diagnostic time is identical to that used in the treatment groups. For time of death, we vary the percent of the brain that is 80% or more necrotic between 3.5% and 4.4% as well as the percent of high density tumor in the brain between 10% and 18%. The time of death criteria for the control group differs from the treatment group because untreated tumor growth is characterized by an expanding high density tumor and necrosis (as opposed to expanding FLAIR and/or necrosis). Table 5 summarizes the key components of our experimental design, including the number of simulated tumors in each group, the motility parameters used to generate each tumor type, the range of variation in treatment, diagnostic, and death times, as well as the median overall survival time found for each group.

### Computing The Spatial Reduction of Mitotic Rate in The Brain

Kirson *et al*. reported on the effects of 24 hours exposure on the rate of proliferation of F-98 rat glioma cells to 200 kHz Tumor Treating Fields of various intensities [36]. The electric field intensity in the culture medium was measured as described [37]. The OptuneTM system (www.Optune.com) is currently being used for clinical application of Tumor Treating Fields in the treatment of glioblastoma multiforme patients [17, 38]. Miranda et al. utilized a realistic head model created from magnetic resonance imaging data to compute the spatial distribution of the induced electric field in the brain. The OptuneTM system was represented in their model, with an applied current of 100 mA at each active transducer [39]. A frequency domain study with 200 kHz was used to compute the electroquasistatic approximation of the Maxwell equations [39]. The calculated spatial distribution was registered onto the head model used for simulating cell proliferation through affine transformation, which was calculated based on user-identified control points.

To translate electric field intensity into a measure of the reduced mitotic rates, a third-degree polynomial was fitted to experimental data points describing proliferation of F-98 rat glioma cells vs. treatment intensity in a Petri dish [36]. This data was used to convert the field intensity matrices derived from [39] into matrices describing the reduction in cell mitotic rate at each point in the head. The field intensity at each point was taken as the maximum of electric fields generated with either the left-right or front-back transducer array. For each point in the brain, the basic mitotic rate was multiplied by the reduction factor to yield the local proliferation rate within the brain.

### Statistics

The Kaplan-Meier statistical analysis of computational trials as well as the calculations of log-rank *p*-values were all performed in Matlab (Mathworks, Natick, MA). Log-rank *p* < 0.05 is the cutoff for statistical significance.
